# Proton pump inhibitors may reduce the efficacy of ribociclib and palbociclib in metastatic breast cancer patients based on an observational study

**DOI:** 10.1186/s12885-022-09624-y

**Published:** 2022-05-07

**Authors:** Kadir Eser, Arif Hakan Önder, Emel Sezer, Timuçin Çil, Ali İnal, Banu Öztürk, Vehbi Erçolak, Berna Bozkurt Duman, Halil Çelik, Tolga Köşeci, Oğuzhan Kesen

**Affiliations:** 1 Faculty of Medicine, Department of Medical Oncology, Mersin University, Çiftlikköy Kampüsü, Yenişehir, 33343 Mersin, Turkey; 2 Department of Medical Oncology, Antalya Education and Research Hospital, Antalya, Turkey; 3 Department of Medical Oncology, Adana Education and Research Hospital, Adana, Turkey; 4 Department of Medical Oncology, Mersin Education and Research Hospital, Mersin, Turkey

**Keywords:** Breast cancer, Ribociclib, Palbociclib, Proton pump inhibitors, PFS

## Abstract

**Introduction:**

Approximately 20–33% of all cancer patients are treated with acid-reducing agents (ARAs), most commonly proton pump inhibitors (PPIs), to reduce gastroesophageal reflux disease symptoms. Palbociclib and ribociclib are weak bases so their solubility depends on different pH. The solubility of palbociclib dramatically decreases to < 0.5 mg/ml when pH is above 4,5 but ribociclibs’ solubility decreases when pH increases above 6,5. In the current study, we aimed to investigate the effects of concurrent PPIs on palbociclib and ribociclib efficacy in terms of progression-free survival in metastatic breast cancer (mBC) patients.

**Patients and methods:**

We enrolled hormone receptor-positive, HER2-negative mBC patients treated with endocrine treatment (letrozole or fulvestrant) combined palbociclib or ribociclib alone or with PPI accompanying our observational study. During palbociclib/ribociclib therapy, patients should be treated with "concurrent PPIs" defined as all or more than half of treatment with palbociclib/ribociclib, If no PPI was applied, it was defined as ‘no concurrent PPI’, those who used PPI but less than half were excluded from the study. All data was collected from real-life retrospectively.

**Results:**

Our study included 217 patients, 105 of whom received palbociclib and 112 received ribociclib treatment. In the study population CDK inhibitor treatment was added to fulvestrant 102 patients ( 47%), to letrozole 115 patients (53%). In the Palbociclib arm fulvestrant/letrozole ratio was 53.3/46.7%, in the ribociclib arm it was 41.07/58.93%. Of 105 patients who received palbociclib, 65 were on concomitant PPI therapy, 40 were not. Of the 112 patients who received ribociclib, 61 were on concomitant PPI therapy, 51 were not. In the palbociclib group, the PFS of the patients using PPIs was shorter than the PFS of the patients not using (13.04 months vs. unreachable, *p* < 0.001). It was determined that taking PPIs was an independent predictor of shortening PFS (*p* < 0.001) in the multivariate analysis, In the ribociclib group, the PFS of the patients using PPIs was shorter than the PFS of the patients not using (12.64 months vs. unreachable, *p* = 0.003). It was determined that taking PPIs was single statistically independent predictor of shortening PFS (*p* = 0.003, univariate analysis).

**Conclusions:**

Our study demonstrated that concomitant usage of PPIs was associated with shorter PFS in mBC treated with both ribociclib and especially palbociclib. If it needs to be used, PPI selection should be made carefully and low-strength PPI or other ARAs (eg H2 antagonists, antacids) should be preferred.

**Supplementary Information:**

The online version contains supplementary material available at 10.1186/s12885-022-09624-y.

## Introduction

Targeted drugs such as tyrosine kinase inhibitors (TKI) and cyclin-dependent kinase (CDK) inhibitors are widely used in cancer patients. These drugs are oral medications, so, gastric pH has a significant effect on drug efficacy. There were many influencing factors on gastric pH, such as feeding and concomitant medications. These drugs can be dissolved well when the appropriate pH is established. Approximately 20–33% of all cancer patients are treated with acid reducing agents (ARAs), most commonly proton pump inhibitors (PPIs), to reduce gastroesophageal reflux disease symptoms. PPIs also interact via the pharmacological and solubility pathways [1, 2]. For this reason, drug-drug interactions (DDIs) at the time of absorption should be considered as one of the causes of treatment failure in cancer patients [3]. In fact, gastric pH elevation by PPIs reduces the oral bioavailability of many drugs used in cancer. This situation is demonstrated to be significant especially in those with exponentially decreasing solubility in the pH range 1–4 [4, 5]. The type of anticancer drugs determines the clinical occurrence of these changes [6]. It has been reported that long-term acid suppression by PPIs reduces the antitumor efficacy of pazopanib and capecitabine, while this effect of PPIs has not been found in clinical outcomes on patients treated with epidermal growth factor receptor (EGFR) tyrosine kinase inhibitors [7–9]. However, there are also studies that show the opposite of these effects [10, 11]. The conflicting results in studies may be due to the fact that PPIs increase susceptibility to gastrointestinal tract-associated infection and induce dysbiosis [12, 13] Since absorption may be affected in dysbiosis, the effectiveness of the drugs may change.

Ribociclib and palbociclib are oral CDK 4/6 inhibitors that arrest the cell cycle by inhibiting DNA synthesis inhibition [14]. The clinical efficacy of the endocrine therapy, either non steroidien aromatase inhibitors and fulvestrant combined with CDK4/6 inhibitors represent a standart of care, for premenopausal or menopausal patients with an estrogene receptor positive, HER2 negative (ER + /HER2-) advanced breast cancer (BC) [15–18]. According to results of randomised controlled trials, when CDK4/6 inhibitor added to letrozole in the first line treatment of HR positive Her2 negative advanced breast cancer patients, median PFS times were doubled and also median OS was improved. Palbociclib is a weak base so its solubility depends on pH. The solubility of palbociclib dramatically decreases to < 0.5 mg/ml when the pH is above 4.5 (i.e. gastric pH typically achieved by PPI). Ribociclib is also a weak base and its solubility decreases when the pH increases above 6.5. Medicines are usually taken with 200–250 ml of water. The in vitro solubility of ribociclib was investigated in biorelevant media consisting of simulated feeding (pH 5.0) and on an empty stomach (pH 6.5) intestinal fluid. The maximum dose of ribociclib (600 mg) was completely dissolved in 250 mL of biorelevant media [19]. Whereas the former palbociclib capsules should not be used with PPI on an empty stomach (C_max_: -80%, AUC: -62%), no significant impact had been seen with coated tablets and PPI. As a consequence, the capsules had to be applied with meals in case of PPI coadministration (with reduction of C_max_) by—41% and AUC by – 13%) [20]. AUC rather c(max) is primarily focused as clinically relevant parameter in DDI regarding PPI and TKI [21]. Clinical trial data and population pharmacokinetics showed that ribociclib absorption was similar at various stomach pH values that occur after food intake or concomitant use of PPIs [19, 22]. According to our knowledge to date, there are insufficient data on DDIs between palbociclib and PPIs other than rabeprazole.

In the current study, we aimed to investigate the effects of concurrent PPIs on palbociclib and ribociclib efficacy in terms of progression free survival in patients with estrogen-positive, HER2-negative metastatic breast cancer (mBC) treated with palbociclib/ribociclib as a first line or subsequent line of treatment.

## Patients and methods

We enrolled hormone receptor-positive, HER2-negative mBC patients treated with palbociclib or ribociclib alone or with PPI accompanying our observational study. Tumors with estrogen receptors in patients with metastatic breast cancer if expression is > 10%, we defined hormone receptor positive as HER2-negative as a score of 0 or 1 + by immunohistochemistry and negative staining by SISH (silver in situ hybridization)/FISH (fluorescent in situ hybridization) in those with a score of 2 + in immunohistochemistry. During palbociclib/ribociclib therapy, patients were treated with "concurrent PPIs" defined as all or more than half of treatment with palbociclib/ribociclib, If no PPI was applied, it was defined as ‘no simultaneous PPI’. Those who used PPIs but less than half were excluded from the study. Based on previous endocrine time response, those with endocrine sensitivity (if relapsed at least 12 months after completion of adjuvant endocrine therapy or de novo metastatic breast cancer) or those who are endocrine resistant (relapse while receiving adjuvant therapy or recurrence within 12 months of discontinuation of adjuvant endocrine therapy and progression within the 6 months after initiating aromatase inhibitor in palliative therapy) [23]. According to the insurance system in our country, it is obligatory to use CDK inhibitor with letrozole in endocrine sensitive patients and with fulvestrant in endocrine resistant patients. Our patients used CDK inhibitors in accordance with this situation.

All clinicians in our study performed pharmacological and clinical interventions in real life according to clinical practice. One course of treatment was given as 28 days, consisting of 21 consecutive days of full and 7 days of blank treatment. Specifically, palbociclib capsules orally at a dose of 125 mg, ribociclib film coated tablets orally at a dose of 600 mg/21 days on and 7 days off), 28-day full cycle plus fulvestrant or letrozole were administrated. Ribociclib dose reduction was made to 400 mg, and palbociclib dose reduction was made to 100 mg based on the toxicity profile. No lower dose was used in any patient. PPIs (lansoprazole 30 mg, esomeprazole 40 mg, omeprazole 40 mg, pantoprazole 40 mg, rabeprazole 20 mg dose) were recommended to take in the morning at breakfast. Ribociclib was used preferably in the morning on an empty or full stomach, and palbociclib with lunch, both at the same time of day..

Strong inhibitors or inducers of cytochrome P450 3A4 (CYP3A4) while taking both drugs were avoided, this management was done according to the individual knowledge of each clinician. The doctors who wrote the prescription followed the patients’ condition in accordance with the recommendations. Toxicity was assessed according to the World Health Organization (WHO) criteria classification. Ethics approval was obtained from the local Institutional Review Board and the Ethic Committee (Ethical Committee of the Faculty of Medicine of the University of Mersin) This retrospective study was performed in compliance with the Declaration of Helsinki.

### Statistical analysis

Eastern Cooperative Oncology Group (ECOG) performance status 0–1 versus ≥ 2, hormone sensitivity versus resistance, premenopausal versus postmenopausal status, CDK inhibitor interval (time between diagnosis of metastasis and initiation of CDK inhibitor treatment) < 18 versus ≥ 18 months, visceral versus bone disease, and the number of tumor sites 1–2 versus > 3 in absolute and median and relative frequencies and quantitative factors are categorical variables. The time from initiation of CDK combination therapy to progression was defined as PFS. For calculating PFS, generating survival curves and log-rank testing, the Kaplan–Meier method was used. Independent risk factors for PFS were determined with the Cox hazard regression method.

## Results

**Fig. 1:**
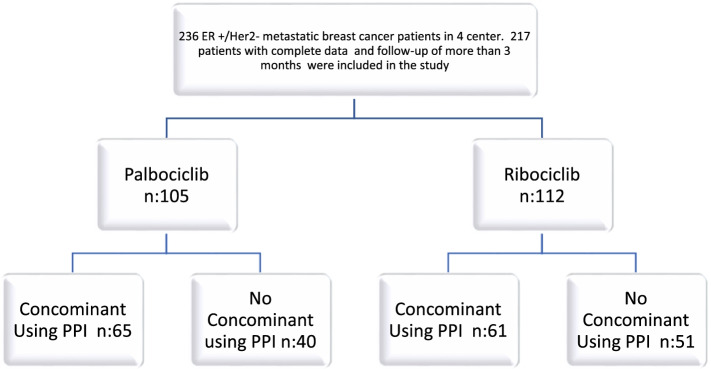
Study profile. ER, estrogene receptor; HER2, human epidermal growth factor receptor 2; PPI, proton pump inhibitor

The files of 236 patients were reviewed, 217 patients with complete data and follow-up of more than 3 months were included in the study. Of these 217 patients, 105 were patients receiving palbociclib and 112 were patients receiving ribociclib.. Of 105 patients who received palbociclib, 65 were on concominant PPI therapy, and 40 were not. Of the 112 patients who received ribociclib, 61 were on concominant PPI therapy, and 51 were not (Fig. 1).

Forty-nine patients received, palbociclib combined with letrozole as first-line endocrine therapy (endocrine sensitive) and 56 endocrine refractory patients used fulvestrant as first- or subsequent line treatment. Of the patients treated with palbociclib, 49 (46.7%) received the 125 mg dose, and 56 (53.3%) received the 100 mg dose. Ribociclib was used as first line endocrine therapy (endocrine sensitive) with letrozol in 66 patients, and combined with fulvestrant as a first or subsequent line of therapy in 46 endocrine refractory patients. For patients treated with ribociclib, 51 (45.6%) patients received the 600 mg dose, and 61 (54.4%) patients received the 400 mg dose. There was no significant difference between the patients who took PPIs and those who did not in either the palbociclib group or the ribociclib group. The clinical characteristics for both drug groups separately are shown in Table 1.

**Table 1:** Clinical characteristics of patients and their distrubution across PPI groups

Characteristic	PALBOCICLIB				RIBOCICLIB			
	Total number of patients (*n* = 105)	Concomitant use of PPIs		P score	Total number of patients (*n* = 112)	Concomitant use of PPIs		P score
		No	Yes			No	Yes	
Age, median (Range)	59 (32–83)	58 (35–76)	61 (32–83)	-	53 (32–87)	49 (32–87)	57 (38–87)	-
Menopausal status, n(%)								
Premenopause	32(30.4)	14(35.0)	18(27.7)	0.514	43(38.3)	22(43.1)	21(34.4)	0.436
Postmenopause	73(69.5)	26(65.0)	47(72.3)		69(61.6)	29(56.9)	40(65.6)	
ECOG PS, n (%)								
0	19(18)	8(20.0)	11(16.9)	0.079	38(33.9)	23(45.1)	15(24.6)	0.072
1	74(70.4)	31(77.5)	43(66.2)		60(53.5)	23(45.1)	37(60.7)	
2	12(11.4)	1(2.5)	11(16.9)		14((12.5)	5(9.8)	9(14.8)	
Disease site, n (%)								
Visceral	63(60)	22(55.0)	41(63.1)	0.421	54(48.2)	24(47.1)	30(49.2)	0.851
Nonvisceral	42(40)	18(45.0)	24(36.9)		58(51.7)	27(52.9)	31(50.8)	
Number of metastatic site, n (%)								
< 3	95(91.5)	37(92.5)	58(89.2)	0.738	98(87.5)	47(92.2)	51(83.6)	0.252
≥ 3	10(9.5)	3(7.5)	7(10.8)		14(12.5)	4(7.8)	10(16.4)	
Endocrine therapy, n (%)								
Letrozole^a^	49(46.6)	23(57.5)	26(40.0)	0.107	66(58.9)	34(66.7)	32(52.5)	0.177
Fulvestrant^a^	56(53.3)	17(42.5)	39(60.0)		46(41)	17(33.3)	29(47.5)	
Dose Reduction, n (%)								
Yes	56(53.3)	19(47.5)	37(56.9)	0.224	61(54.4)	25(49.0)	36(59.0)	0.254
No	49(46.7)	21(52.5)	28(43.1)		51(45.6)	26(51.0)	25(41.0)	
CDK inhibitor interval^b^								
< 18 months	65(61.9)	27(67.5)	38(58.5)	0.411	86(76.7)	41(80.4)	45(73.8)	0.502
≥ 18 months	40(38)	13(32.5)	27(41.5)		26(23.2)	10(19.6)	16(26.2)	
PPI, n (%)								
Pantoprazole			28(43.1)				18(29.5)	
Rabeprazole			17(26.2)				10(16.4)	
Esomeprazole			8(12.3)				18(29.5)	
Lansoprazole			9(13.8)				3(4.9)	
Omeprazole			3(4.6)				12(19.7)	

^a^All hormone sensitive patients used letrozole + CDK inhibitor combination, all hormone resistant patients used fulvestrant + CDK inhibitor combination

^b^CDK interval: Time between metastatic breast cancer (MBC) diagnosis and CDK treatment

**Fig. 2:**
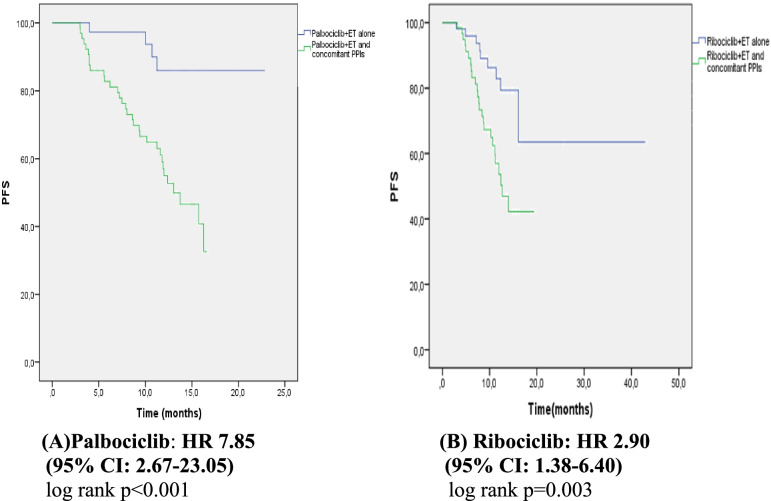
PFS curves of Palbociclib and Ribociclib combined endocrine therapy with or without PPIs. (Kaplan meier estimates). ET: endocrine treatment, PFS: progression free survival, PPI: proton pomp inhibitör, CI: confidence interval, HR: hazard ratio

**Fig. 3:**
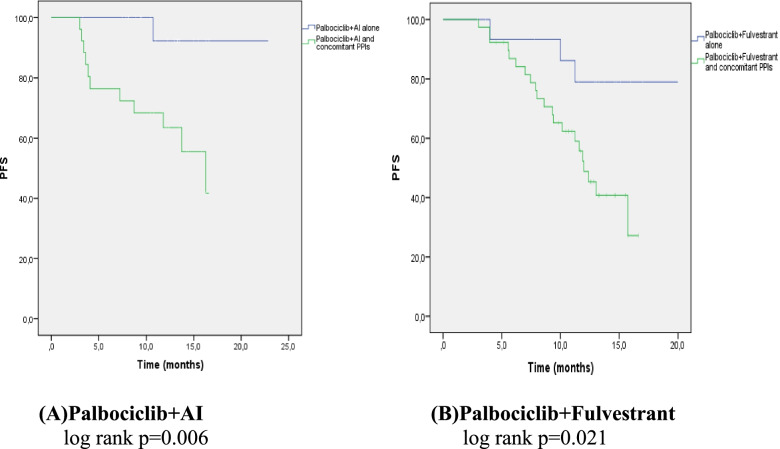
PFS curves of Palbociclib + AI and Palbociclib + Fulvestrant with or without PPIs. (Kaplan meier estimates) AI: aromatase inhibitor, PFS: progression free survival, PPI: proton pomp inhibitör

In the palbociclib group, the PFS of the patients using PPIs was shorter than the PFS of the patients not using PPIs (13.04 months vs. unreachable, *p* < 0.0001, respectively; Fig. 2A). After a mean follow-up of 13 months, 83% of the patients who did not take PPIs did not progress. Univariate analysis included age, CDK combination, number of metastatic sites, ECOG, menopausal status, dose reduction, metastasis diagnosis time, and CDK starting interval (CDK interval). Age, number of metastatic sites, ECOG PS, and menopausal status were found to be significantly associated with PFS (*p* = 0.001, *p* = 0.006, *p* = 0.048, *p* = 0.008, respectively; Table 2). As a result of multivariate analysis, it was determined that taking PPIs was an independent predictor of shortening PFS (hazard ratio 5.60; 95% confidence interval: 1.98–15.85; *p* =  < 0.001; Table 2). When we analysed the effective role of PPI use on PFS separately in the letrozole (hormone sensitive) and fulvestrant (hormone resistant) groups, the PFS was significantly shorter in patients using PPIs in both groups ( *p* = 0.006, *p* = 0.021 Fig. 3A. B).

**Table 2:** Univariate and multivariate analysis for progression-free survival

Variables	PALBOCICLIB				RIBOCICLIB			
	Univariate		Multivariate		Univariate		Multivariate	
	HR (95%) Cl	*P* value	HR (95%) Cl	*P* value	HR (95%) Cl	*P* value	HR(95%) Cl	*P* value
Age	0,94 (0,91–0,97)	**0,001**	0.95(0.90–1.00)	0.053	0,99 (0.96–1.02)	0.758		
Number of metastatic sites	3,8 (1.46–10.04)	**0.006**	2.28 (0.64–8.09)	0.201	0.95 (0.56–1.59)	0.403		
CDK inhibitor combination	1.8 (0.91–3.67)	0.089			1.26 (0.63–2.48)	0.509		
ECOG PS	0.215 (0.04–0.98)	**0.048**	0.66 (0.09–4.95)	0.694	0.67 (0.21–2.09)	0.769		
Pre/Post-menopause	0.394	**0.008**	0.72 (0.25–2.03)	0.537	0.71 (0.36–1.41)	0.340		
Visseral-nonvisseral disease	0.58 (0,28–1.11)	0.130			0.59 (0.29–1.18)	0.135		
Dose reduction	1.22 (0.62–2.37)	0.550			1.21 (0.60–2.44)	0.587		
CDK inhibitor interval	1.92 (0.99–3.71)	0.054			1.41 (0.68–2.89)	0.361		
Concomitant use of PPIs	5.60 (1.98–15.85)	** < 0.001**	7.85 (2.67–23.05)	** < 0.001**	2.90 (1.38–6.40)	**0.003**	2.90 (1.38–6.40)	**0.003**

**Fig. 4:**
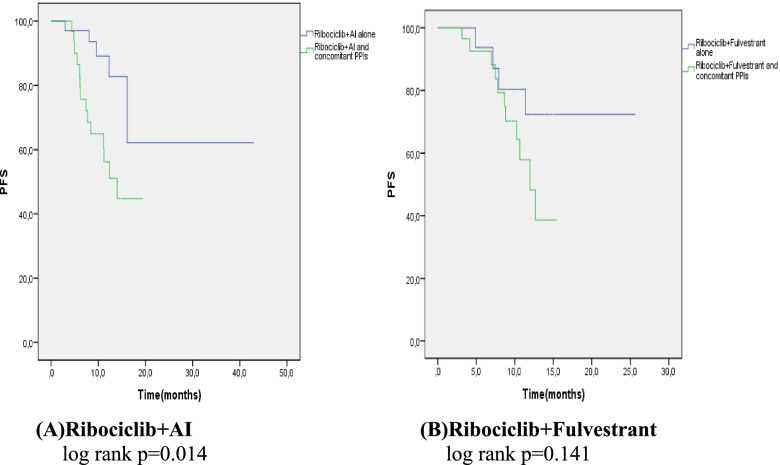
PFS curves of Ribociclib + AI and Ribociclib + Fulvestrant with or without PPIs. (Kaplan meier estimates) AI: aromatase inhibitor, PFS: progression free survival, PPI: proton pomp inhibitör

In the ribociclib group, the PFS of the patients using PPIs was shorter than the PFS of the patients not using PPIs (12.64 months vs. unreachable, *p* = 0.003, respectively; Fig. 2B). After a mean follow-up of 15 months, 65% of the patients who did not take PPIs did not progress. Univariate analysis included age, CDK combination, number of metastatic sites, ECOG, menopausal status, dose reduction, metastasis diagnosis time, and CDK starting interval (CDK interval). No statistical significance was found in any of the univariate analyses. Only PPI use was found to have a significant effect on PFS in patients receiving ribociclib (hazard ratio 2.9; 95% confidence interval: 1.38–6.40;*p* = 0.003; Table 2). When we analysed the effective role of PPI use on PFS separately in the letrozole (hormone sensitive) and fulvestrant (hormone resistant) groups, the PFS was significantly shorter in patients using PPIs in the letrozole group (*p* = 0.014, *p* = 0.141 Fig. 4A, B).

The patients who participated in the study did not have intolerance to completely discontinue the drug, but dose reduction was performed in some patients as a result of grade 3–4 side effects. In both the palbociclib group and the ribociclib group, there was no statistically significant difference in grade 3–4 adverse events requiring dose reduction between the patient groups taking and not taking PPIs (*p* = 0.224, *p* = 0.254; Table 1).

## Discussion

Among different factors such as fast, feeding, concomitant drugs, gastric pH increase, etc., the pH solubility of the drug is considered to be the most relevant influencing drug absorption [24]. When stomach pH increases, the effectiveness of oral anticancer drugs with weak base properties decreases due to decreased bioavailability [4, 25]. To our knowledge, our study was the first to show that concomitant usage of PPIs with palbocicilib/ribociclib in patients with mBC had a detrimental effect on PFS. We concluded that increasing gastric pH induced by PPIs may occur through lowering palbociclib plasma concentrations, which affects treatment efficacy and results in shorter progression-free survival. Palbociclib is a weak pH-dependent base with gradually increasing solubility when the pH rises above 4.5. Rabeprazole-induced changes in post-fed status on palbociclib pharmacokinetics were not considered clinically significant, and no restrictions for concomitant use of PPIs have been reported in palbociclib labelling [20, 26]. However, the clinical consequences of rabeprazole’s ability to reduce efficacy it were not investigated in the study performed by Sun et al. [20]. Additionally, while investigating the effect of rabeprazole on palbociclib pharmacokinetics, giving just 6 days may not have been enough, because in short-term treatment with PPIs, intragastric pH may not be increased throughout the 24-h interval [18, 27]. In our study, PPIs (mainly pantoprazole, rabeprazole, esomeprazole) were given at no less than half of all palbociclib therapy for a greater and steady rise in intragastric pH.

The PFS of palbociclib with letrozole in paloma 2 and fulvestrant in paloma 3 was 27.6 months and 9.2 months, respectively, and the PFS of ribociclib with letrozole in monaleesa 2 and fulvestrant in monaleesa 3 was 25.3 months and 20.5 months, respectively [15–18]. According to our evaluation, the reason why PFS was lower in paloma 3 than in monaleesa 3 was that all patients were endocrine resistance and some patients who received 1 step of chemotherapy in paloma 3 were included in the study, while those endocrine resistance or sensitive patients were included in monaleesa 3 and who received chemotherapy in monaleesa 3 were not included in the study. In our study, some of the patients who received letrozole combination or fulvestrant combination had a history of chemotherapy in metastatic disease; therefore, the PFS of our study may be shorter. In the study by Re et al., PFS was 14 months versus 37.9 months in patients who received and did not receive concominant PPIs with palbociclib, respectively. Additionally, no other significant variable affecting PFS was detected in the multivariate analysis [28]. In the results we presented, PFS was similar to that in this trial in patients who received PPIs, but PFS could not be reached yet in patients who did not receive PPIs.

When below the absolute threshold level, although it is not known at this time that the activity of palbociclib may be affected, palbociclib cell potency in vitro (IC_50_) with free mean steady-state concentration (C_ss_) is comparable with a C_ss_/IC_50_ ratio of 0.94 [29]. The findings of the present study support the following hypothesis: prolonged treatment with PPIs may reduce palbociclib to plasma levels below the threshold of the minimum effective concentration, thus reducing its effectiveness to some extent. Failure to evaluate the pharmacokinetic changes induced by PPIs in palbociclib is a limitation of our study. Additionally, studies have shown that short-term treatment with rabeprazole reduces fasting palbociclib Cmax by 80% and 41% at fasting and fed, respectively [20].

There is a little evidence in the literature suggesting that agents that alter gastric pH have no effect on ribociclib absorption [19, 22]. Samant et al. examined the steady-state pharmacokinetics of ribociclib (600 mg) during PPI use and found no differences in AUC and C_max_ between the PPI-using and non-PPI-using groups [19]. However, that is not specified in this study is whether these patients used the drug when they were on an empty stomach or when they were full. The different behaviors of ribociclib and palbociclib in acidic media may be due to the difference in their dissolution strength. Consistent with this information, the solubility of ribociclib is > 2.4 mg/ml at pH 4.5 and 0.8 mg/ml at pH > 6.8, while that of palbociclib is > 0.5 mg/ml at pH < 4.5 only [19, 20]. Examining the in vitro solubility of ribociclib by simulating fasting intestinal fluid (pH 6.5) and postprandial intestinal fluid (pH 5.0) in biorelevant madia, 600 mg was dissolved in 250 ml of fluid [19].This feature of ribociclib makes it less affected by PPIs, but its absorption may be affected in environments where stomach acid is potently inhibited, especially in fasting conditions. Therefore, it may be more beneficial to take ribociclib with meals in patients taking ribociclib plus PPIs. Saman et al. reported that trough concentration mean ribociclib values ​​(C_trough_) were 597 and 711 ng/ml in patients with or without PPI at 600 mg dose, respectively [19]. On average, free C_ss_ expressing a broad therapeutic index a reduction in ribociclib C_trough_, is unlikely, as it greatly exceeds in vitro cell potency (C_ss_/IC_50_ ratio > 25) [29]. But in real life, almost half of the patients use Ribociclib at a dose of 400 mg. Therefore, C_trough_ values ​​may fall below effective levels. While ribociclib was not expected to be affected by PPI according to its pharmacokinetic and pharmacodynamic properties, it was the independent factor affecting PFS in our study. One of the reasons for this may be the induction of dysbiosis and increased risk of gastrointestinal tract infection [12, 13]. In addition to the absorption and pH change mechanisms of PPI, one of the mechanisms that reduces palbociclib capsule effectiveness is likely to be the induction of dysbiosis. With respect to abemaciclib, this drug also shows pharmacokinetic similarities when compared to other CDK4/6 inhibitors. Notable features are saturable absorption with twice daily administration due to smaller volume of distribution and shorter half-life than ribociclib and palbociclib [30].

It remains unclear whether PgP or other pumps are clinically relevant regarding PPI – related DDI. According to our knowledge, palbociclib and ribociclib are P-gp substrates and are moderately inhibited by PPIs [31, 32]. Additionally, TKI pharmacokinetics were found to be altered by pantoprazole through the influence of breast cancer resistance protein (BCRP) and P-gp [27]. If the main mechanism of DDI is P-gp had it been inhibited by PPIs, fewer side effects would have been expected in PPI users due to the effect caused by the increase in gastric pH. In the presence or absence of PPIs, as the differences in adverse drug reactions were not statistically significant, so this hypothesis is not compatible with our data. Accordingly, rabeprazole is known to inhibit P-gp activity at appropriate concentrations, and its clinical net effect reduces palbociclib exposure [20]. However, this effect is great at fasting, in environments where the pH is higher. Therefore, gastric pH changes due to PPIs appear to be the main mechanism of interaction with drugs that require an acidic microenvironment for dissolution and absorption [33].

Studies to date have reported other instances of DDIs between PPIs and TKIs (i.e. pazopanib, sunitinib, gefitinib, and erlotinib) [8, 11, 34–38]. A meta-analysis of 16 retrospective studies involving various solid tumors with a total of 372,418 patients demonstrated that PPI therapy had a significant impact on survival outcomes in patients receiving oral anticancer drugs [39]. The effect of concomitant PPI administration on overall survival and treatment discontinuation, 90 days and 1 year after discontinuation, on overall survival in another 12 538 patients retrospective study with solid and haematological tumours evaluated. This study was performed retrospectively in patients treated with TKIs, and PPI use has been shown to be associated with an increased risk of death [40].

There were some limitations of our study. First, the adverse event profile can be underestimated because of the retrospective nature of our study. However, in the current study, dose reductions of CDK inhibitors were performed more than in other clinical trials. We generally used CDK inhibitors in the COVID-19 pandemic because the labelling time of palbociclib and ribociclib by health authorities in our country was May 2020, so physicians are sensitive to dose reduction when grade 3–4 neutropenia develops. Despite these limitations, we collected soluble and reliable data with satisfactory sample sizes. It was clearly demonstrated that concomitant usage of PPIs was associated with shorter PFS. We recommend caution in the long-term use of PPIs in this specific population and the benefits-risks of coadministration of anticancer drugs whose solubility and absorption depend on pH and strong acid-reducing agents should be evaluated and decided together. If used, PPI selection should be made carefully. For example, rabeprazole may provide more and longer acid suppression than other drugs in the same class; in treatment management H2-antagonists should also be considered instead of PPIs. Increasing the dose of palbociclib in patients using PPIs may theoretically make sense, but in clinical practice it is probably not an effective strategy due to possible off-label effects. If it is necessary to use PPI together with ribociclib, it should be used on a fed.

## Supplementary Information

**Additional file 1. **Statistical analyses. Ki square tests, Kaplan Meier Test, Cox hazard regression was made using regarding these data. 

## Data Availability

The datasets generated and/or analysed during the current study are not publicly available due [reason, personal data protection law] but are available from the corresponding author on reasonable request.

## Abbreviations

TKI: Tyrosine kinase inhibitor
CDK: Cyclin dependent kinase
ARAs: Acid reducing agents
PPIs: Proton pump inhibitors
DDI: Drug drug interactions
EGFR: Epidermal growth factor receptor
HER2: Human epidermal growth factor receptor 2
AUC: Area under the concentration time curve
C_max_: Maximum plasma concentration
mBC: Metastatic breast cancer
SISH: Silver in situ hybridization
FISH: Fluorescent in situ hybridization
WHO: World health organization
ECOG: Eastern Cooperative Oncology Group (ECOG)
IC_50_: In vitro cell potency
C_ss_: Steady-state concentration
C_trough_: Trough concentration
P-gp: P-glycoprotein
BRCP: Breast cancer resistance protein

## References

[1] Smelick GS (2013). Prevalence of acid-reducing agents (ARA) in cancer populations and ARA drug–drug interaction potential for molecular targeted agents in clinical development. Mol Pharm.

[2] Raoul JL (2021). Prevalence of proton pump inhibitor use among patients with cancer. JAMA Netw Open.

[3] Wedemeyer R-S, Blume H (2014). Pharmacokinetic drug interaction profiles of proton pump inhibitors: an update. Drug Saf.

[4] Budha NR (2012). Drug absorption interactions between oral targeted anticancer agents and PPIs: is pH-dependent solubility the achilles heel of targeted therapy?. Clin Pharmacol Ther.

[5] Mudie DM (2014). Quantification of gastrointestinal liquid volumes and distribution following a 240 mL dose of water in the fasted state. Mol Pharm.

[6] Riechelmann RP, Krzyzanowska MK (2019). Drug interactions and oncological outcomes: a hidden adversary. Ecancermedicalscience.

[7] Hilton JF (2013). An evaluation of the possible interaction of gastric acid suppressing medication and the EGFR tyrosine kinase inhibitor erlotinib. Lung Cancer.

[8] Kumarakulasinghe NB (2016). EGFR kinase inhibitors and gastric acid suppressants in EGFR-mutant NSCLC: a retrospective database analysis of potential drug interaction. Oncotarget.

[9] Vishwanathan K (2018). The effect of food or omeprazole on the pharmacokinetics of osimertinib in patients with non-small-cell lung cancer and in healthy volunteers. J Clin Pharmacol.

[10] Cheng V (2019). Concomitant use of capecitabine and proton pump inhibitors - Is it safe?. J Oncol Pharm Pract.

[11] Zenke Y (2016). Clinical impact of gastric acid-suppressing medication use on the efficacy of erlotinib and gefitinib in patients with advanced non–small-cell lung cancer harboring EGFR mutations. Clin Lung Cancer.

[12] Corley DA (2019). Safety and Complications of Long-Term Proton Pump Inhibitor Therapy: Getting Closer to the Truth. Gastroenterology.

[13] Moayyedi P (2019). Safety of proton pump inhibitors based on a large, multi-year, randomized trial of patients receiving rivaroxaban or aspirin. Gastroenterology.

[14] Spring LM (2020). Cyclin-dependent kinase 4 and 6 inhibitors for hormone receptor-positive breast cancer: past, present, and future. Lancet.

[15] Finn RS (2016). Palbociclib and letrozole in advanced breast cancer. N Engl J Med.

[16] Hortobagyi GN (2018). Updated results from MONALEESA-2, a phase III trial of first-line ribociclib plus letrozole versus placebo plus letrozole in hormone receptor-positive, HER2-negative advanced breast cancer. Ann Oncol.

[17] Slamon DJ (2018). Phase III randomized study of ribociclib and fulvestrant in hormone receptor-positive, human epidermal growth factor receptor 2-negative advanced breast cancer: MONALEESA-3. J Clin Oncol.

[18] Turner NC (2018). Overall survival with palbociclib and fulvestrant in advanced breast cancer. N Engl J Med.

[19] Samant TS (2018). Ribociclib bioavailability is not affected by gastric pH changes or food intake: in silico and clinical evaluations. Clin Pharmacol Ther.

[20] Sun W (2017). Impact of acid-reducing agents on the pharmacokinetics of palbociclib, a weak base with pH-dependent solubility, with different food intake conditions. Clin Pharmacol Drug Dev.

[21] Hussaarts K (2019). Clinically relevant drug interactions with multikinase inhibitors: a review. Ther Adv Med Oncol.

[22] Lu Y (2021). Ribociclib population pharmacokinetics and pharmacokinetic/pharmacodynamic analysis of neutrophils in cancer patients. J Clin Pharmacol.

[23] Cardoso F (2018). 4th ESO–ESMO international consensus guidelines for Advanced Breast Cancer (ABC 4)††these guidelines were developed by the European School of Oncology (ESO) and the European Society for Medical Oncology (ESMO). Ann Oncol.

[24] Willemsen AECAB (2016). Effect of food and acid-reducing agents on the absorption of oral targeted therapies in solid tumors. Drug Discov Today.

[25] van Leeuwen RWF (2014). Drug–drug interactions with tyrosine-kinase inhibitors: a clinical perspective. Lancet Oncol.

[26] IBRANCE®, Full Prescribing Information. 2016.

[27] van Leeuwen RWF (2017). Tyrosine kinase inhibitors and proton pump inhibitors: an evaluation of treatment options. Clin Pharmacokinet.

[28] Del Re M (2021). Drug-drug interactions between palbociclib and proton pump inhibitors may significantly affect clinical outcome of metastatic breast cancer patients. ESMO open.

[29] Goldstein MJ (2021). Optimizing the therapeutic window of targeted drugs in oncology: potency-guided first-in-human studies. Clin Transl Sci.

[30] Braal CL (2021). Inhibiting CDK4/6 in breast cancer with palbociclib, ribociclib, and abemaciclib: similarities and differences. Drugs.

[31] de Gooijer MC (2015). P-glycoprotein and breast cancer resistance protein restrict the brain penetration of the CDK4/6 inhibitor palbociclib. Invest New Drugs.

[32] Martínez-Chávez A (2019). P-glycoprotein limits ribociclib brain exposure and CYP3A4 restricts its oral bioavailability. Mol Pharm.

[33] Ollier E (2015). In vitro and in vivo evaluation of drug–drug interaction between dabigatran and proton pump inhibitors. Fundam Clin Pharmacol.

[34] Fang Y-H (2019). Concurrent proton-pump inhibitors increase risk of death for lung cancer patients receiving 1st-line gefitinib treatment-a nationwide population-based study. Cancer Manag Res.

[35] Ha VH (2014). Does gastric acid suppression affect sunitinib efficacy in patients with advanced or metastatic renal cell cancer?. J Oncol Pharm Pract.

[36] Lalani A-KA (2017). Proton pump inhibitors and survival outcomes in patients with metastatic renal cell carcinoma. Clin Genitourin Cancer.

[37] McAlister RK (2018). Effect of concomitant pH-elevating medications with pazopanib on progression-free survival and overall survival in patients with metastatic renal cell carcinoma. Oncologist.

[38] Veerman GDM (2021). Influence of cow’s milk and esomeprazole on the absorption of erlotinib: a randomized, crossover pharmacokinetic study in lung cancer patients. Clin Pharmacokinet.

[39] Indini A (2020). Impact of use of gastric-acid suppressants and oral anti-cancer agents on survival outcomes: a systematic review and meta-analysis. Cancers.

[40] Sharma M (2019). The concomitant use of tyrosine kinase inhibitors and proton pump inhibitors: prevalence, predictors, and impact on survival and discontinuation of therapy in older adults with cancer. Cancer.

