# Supplementary material for: Proton pump inhibitors may reduce the efficacy of ribociclib and palbociclib in metastatic breast cancer patients based on an observational study
Source: BMC Cancer. 2022 May 7;22:516. doi: 10.1186/s12885-022-09624-y (PMC9078089; doi:10.1186/s12885-022-09624-y)
Supplement: Supplementary file 1 — Additional file 1. Statistical analyses. Ki square tests, Kaplan Meier Test, Cox hazard regression was made using regarding these data. [file 12885_2022_9624_MOESM1_ESM.zip › Supplementary file.docx]

SUPPORTİNG FİLE

**Table1**.Impact of PPI to CDK formulations with on an empty stomach versus with fat-containing meals

| Impact of PPI | Palbociclib capsules | Palbociclib (coated tablets) | Ribociclib (coated tablets) |
| --- | --- | --- | --- |
| Intake on an empty stomach | AUC: -62%, c(max): -80% | no impact | no impact |
| Intake with fat- containing meals | AUC: -13%, c(max): -41% | no impact | no impact |
